# Microbial Community Structure of Mesophilic and Low-temperature Partial Nitrification-anammox Reactors: Distribution and Functional Roles of the Core Microbiome

**DOI:** 10.1264/jsme2.ME25001

**Published:** 2025-04-26

**Authors:** Mamoru Oshiki, Kohei Takahashi, Seiya Kawasaki, Hyungmin Choi, Jihye Park, Kwiyong Kim, Hyokwan Bae, Satoshi Okabe, Changsoo Lee

**Affiliations:** 1 Division of Environmental Engineering, Faculty of Engineering, Hokkaido University, Sapporo, Japan; 2 JSPS Postdoctoral Fellow, Hokkaido University, Japan; 3 Department of Civil Urban Earth and Environmental Engineering, Ulsan National Institute of Science and Technology (UNIST), Republic of Korea; 4 Department of Civil and Environmental Engineering, Pusan National University, Republic of Korea; 5 Graduate School of Carbon Neutrality, Ulsan National Institute of Science and Technology (UNIST), Republic of Korea

**Keywords:** anammox, partial nitrification, microbial community structure, *Sulfurisoma*, *Zeimonas*

## Abstract

Microbial community structures in mesophilic and low-temperature anammox and partial nitrification–anammox reactors were exami­ned by a 16S rRNA–gene amplicon sequencing ana­lysis. The anammox bacterium, *Jettenia* sp., was dominant, and nitrifying bacteria, including *Nitrosomonas* sp. (aerobic ammonia–oxidizing bacterium) and *Nitrospira* sp., (nitrite–oxidizing bacterium) coexisted in the bioreactors. Core coexisting bacteria, such as *Sulfurisoma* sp. and *Zeimonas* sp., showed oxygen-scavenging and NO_3_^–^ reduction potentials. *Sulfurisoma*-related bacteria are distributed across wastewater treatment plants worldwide, particularly in denitrification systems. These results underscore the ecological and functional importance of microbial consortia in enhancing nitrogen removal efficiency.

Anaerobic ammonium oxidation (anammox) is an eco-friendly biological nitrogen removal technology that utilizes nitrite (NO_2_^–^) supplied by partial nitrification (PN) or partial denitrification as an electron acceptor to oxidize ammonium (NH_4_^+^) to dinitrogen (N_2_) gas (Strous *et al.*, 1999). The anammox process significantly reduces energy consumption for aeration (by approximately 60%), excess sludge production (by 80–90%), and the need for external organic carbon addition for denitrification, resulting in substantial cost savings and improved energy efficiency in wastewater treatment systems (Ali & Okabe, 2015; Jetten *et al.*, 2001). Although full-scale single– and two–stage PN–anammox processes have been successfully applied to high-strength wastewater, such as livestock wastewater and reject water containing NH_4_^+^ at several hundred mg NH_4_^+^-N L^–1^ (Lackner *et al.*, 2014; Ali and Okabe, 2015), their application to low-strength wastewater, such as municipal sewage (NH_4_^+^ available at several tens of mg NH_4_^+^-N L^–1^), and operation at low temperatures (*e.g.*, 8–20ºC in cold regions and/or during the winter season) remain challenging (Cao *et al.*, 2017; Wang *et al.*, 2022). In low-strength wastewater, difficulties are associated with suppressing the growth of NO_2_^–^-oxidizing bacteria (NOB) and maintaining a sufficient supply of NO_2_^–^ for the anammox process. Additionally, microbial activity generally decreases under low-temperature conditions, leading to a reduction in nitrogen removal rates (Hendrickx *et al.*, 2012; Hu *et al.*, 2013). To overcome these limitations and optimize the configuration of biological nitrogen removal using the anammox process, a more detailed understanding of the microbial ecology involved in anammox and PN processes is essential. However, the microbial community structure in anammox and PN-anammox reactors has not yet been fully exami­ned, particularly in bioreactors fed with low-strength wastewater and operated under low-temperature conditions. Therefore, the present study investigated the microbial community structures and core bacterial genera of anammox reactors and a PN–anammox reactor operated under different configurations and conditions by a 16S rRNA gene-amplicon sequencing ana­lysis. Furthermore, the distribution and abundance of the identified core bacterial genera coexisting with anammox bacteria were exami­ned in wastewater treatment plants worldwide using the MiDAS4 database (Dueholm *et al.*, 2022).

Sludge biomass was collected from three laboratory-scale anammox reactors and one PN–anammox reactor (Table 1). Two anammox reactors (AN–M1 and AN–M2, respectively) were operated under mesophilic conditions (33 or 37°C), while the remaining anammox reactor and the PN–anammox reactors (AN–L1 and PN–AN–L2, respectively) were operated at 10 or 7°C, respectively. Sludge biomass collected from a lab-scale PN–anammox reactor (Jo *et al.*, 2020) was inoculated into the AN–M1 and AN–L1 reactors, whereas the biomass collected from a pilot scale PN–anammox reactor fed with reject water in Daegu was used as an inoculum for the AN–M2 and PN–AN–L2 reactors. Aeration was performed in the PN–AN–L2 reactor to supply dissolved oxygen (DO) required for PN, and the DO concentration was maintained at <0.5‍ ‍mg L^–1^ using a DO controller. Synthetic wastewater containing NH_4_^+^ (30–175‍ ‍mg N L^–1^) (the detailed composition is available in Table S1) was fed into the bioreactors, and operated for >100 days under stable operational conditions. The anammox and PN–anammox reactors showed stable nitrogen removal performance, with NH_4_^+^ removal efficiencies >76% and nitrogen removal efficiencies >60%. In the PN–AN–L2 (PN-anammox) reactor, 18% of influent NH_4_^+^ was fully oxidized to NO_3_^–^, which resulted in lower nitrogen removal efficiency than the other anammox reactors.

Genomic DNA was extracted from sludge samples by the bead beating method, and subjected to the PCR amplification of the 16S rRNA gene using the oligonucleotide primers 515F (5′-GTGCCAGCMGCCGCGGTAA-3′) and 806R (5′-GGACTACHVGGGTWTCTAAT-3′) (Caporaso *et al.*, 2012). PCR amplicon was subjected to 300-bp paired-end sequencing using Illumina MiSeq. Raw sequence reads‍ ‍were subjected to quality filtering using the fastx_barcode_splitter tool from FASTX-Toolkit (ver. 0.0.14), and 70,679–84,650 paired sequence reads per sample were analyzed using QIIME2 software (ver. 2024.2) (Caporaso *et al.*, 2010). Reads were clustered into amplicon sequence variants (ASVs) using the DADA2 plugin (Callahan *et al.*, 2016), and the phylogeny of ASVs was exami­ned using the blastn (ver. 2.9.0) program against the Greengene (ver. 13_8) and nr (accessed on 16^th^ December 2024) databases. The metabolic potential of ASVs was predicted using PICRUSt2 software (Douglas *et al.*, 2020) and also by the manual genome annotation of closely-related species using the KAAS (Moriya *et al.*, 2007) and DRAM annotation tools (Shaffer *et al.*, 2020).

A total of 140 ASVs (>0.2% of relative abundance in at least one sludge biomass sample) were found in the bioreactors exami­ned (Fig. 1a). Dominant ASVs were affiliated into the bacterial phyla *Planctomycetota* (including anammox bacteria), *Pseudomonadota*, *Chloroflexota*, *Chlorobiota*, and *Nitrospirota*. Detailed phylogenetic affiliations of the dominant ASVs, including functional microbial groups (*i.e.*, anammox, aerobic ammonia–oxidizing bacteria [AOB] and NOB), are shown in Fig. S1. *Jettenia* sp. ASV326 was the dominant anammox bacterium (26.4–50.9% in total biomass), and other anammox bacteria related to the genera *Brocadia* and *Anammoxoglobus* coexisted in the AN–M2 and PN–AN–L2 reactors. The bacterial genus *Candidatus Jettenia* represents a lineage of freshwater anammox bacteria with a physiological temperature range of 20–42.5°C (Ali *et al.*, 2015). The presence and distribution of *Jettenia* sp. ASV326 in the low–temperature bioreactors (AN–L1 and PN–AN–L2) suggested that this bacterium is capable of acclimating to low-temperature conditions, as previously reported by a proteomic study (Lin *et al.*, 2018). However, the adaptation mechanisms of *Jettenia* bacteria to low temperatures remain unclear (Kouba *et al.*, 2022), and warrant further study. *Nitrosomonas* sp. ASV917 (99.6% sequence identity with the *Nitrosomonas europaea* ATCC25978 16S rRNA gene) is an AOB that was abundant in the low-temperature bioreactors (0.9 and 2.5% of the total biomass in the AN–L1 and PN–AN–L2 reactors, respectively). The high abundance of *Nitrosomonas* sp. ASV917 in the PN–AN–L2 reactor indicated that this bacterium was responsible for PN and supplying NO_2_^–^ to *Jettenia* sp. ASV326. *Nitrospira* bacteria (ASV496, ASV498, and ASV499) are canonical NOB, whereas the sequence read affiliated into the phylogenetic clade of complete ammonia oxidation (comammox) *Nitrospira* (Daims *et al.*, 2015; van Kessel *et al.*, 2015) was not detected in the bioreactors exami­ned. Phylogenetically diverse NOB *Nitrospira* clades have been described, exhibiting a wide range of physiological characteristics, such as affinity for and tolerance to NO_2_^–^ (Fujitani *et al.*, 2013; Ushiki *et al.*, 2013). *Nitrospira* sp. ASV496 found in the AN–M2 and PN–AN–L2 reactors (1.0 and 1.8%, respectively) related to *Nitrospira tepida* DNF (100% of sequence identity). *N. tepida* has been characterized as a moderately thermophilic bacterium with an optimal growth temperature range of 37–45°C (Keuter *et al.*, 2023), and the present study expanded its known temperature range by 7°C, as observed in the PN–AN–L2 reactor. Additionally, *Nitrospira* bacteria prefer microaerobic conditions over fully aerobic conditions (Lücker *et al.*, 2010), and the DO concentration in the PN–AN–L2 reactor (<0.5‍ ‍mg L^–1^) is favorable for their proliferation. The overgrowth of *Nitrospira* sp. is detrimental to the nitrogen removal efficiency of the PN–anammox process because these bacteria oxidize NO_2_^–^, a substrate of anammox bacteria, to NO_3_^–^, thereby reducing the availability of NO_2_^–^. Therefore, the growth of the detected *Nitrospira* sp. ASVs needs to be suppressed in order to improve the nitrogen removal performance of the PN–AN–L2 reactor.

A principal component ana­lysis (PCA) was performed to examine similarities in microbial community structures among the bioreactors (Fig. 1b). The PC1 axis accounted for a cumulative contribution of 92.7%, and the microbial community structures in the low-temperature bioreactors (AN–L1 and PN–AN–L2) were distinct from those in the mesophilic bioreactors (AN–M1 and AN–M2) along the PC1 axis. This differentiation showed the impact of temperature in shaping anammox bacterial community structures (Sonthiphand *et al.*, 2014; Oshiki *et al.*, 2016). To identify a core microbiome shared across all temperature and operational conditions, the mean relative abundance and coefficient of variation (CV) values were calculated in the sludge samples exami­ned (Fig. 1c). The following 5 ASVs showed high abundance and wide distributions: *Jettenia* sp. ASV326, *Sulfurisoma* sp. ASV867 (classified as the genus Dok59 in the Greengene database ver. 13_8), *Zeimonas* sp. ASV884, *Phycisphaerales* sp. ASV505, and *Anaerolineae* sp. ASV027. *Phycisphaerales* and *Anaerolineae* bacteria have often been detected as coexisting bacteria in anammox bacterial cultures and their potential function (*e.g.*, the degradation of extracellular polymeric substances [EPS]) has been exami­ned using metagenomic ana­lyses (Speth *et al.*, 2016; Lawson *et al.*, 2017; Ali *et al.*, 2020; Oshiki *et al.*, 2022), whereas limited information is available for *Sulfurisoma* sp. ASV867 (97.6% identity to *Sulfurisoma sediminicola* BSN1) and *Zeimonas* sp. ASV884 (98.4% identity to *Zeimonas arvi* CC–CFT501). *S. sediminicola* and *Z. arvi* were isolated from freshwater lake sediment and a maize field as a sulfur-oxidizing bacterium and a bacterium harboring biphenyl- and phenolic acid-metabolizing genes, respectively (Kojima and Fukui, 2014; Lin *et al.*, 2021). As shown in Fig. S2, the *S. sediminicola* BSN1 and *Z. arvi* CC–CFT501 genomes (accession numbers: GCF_003865015.1 and GCF_008039575.1, respectively) contain the genes involved in aerobic respiration (including terminal cytochrome *c* oxidases, such as high-affinity *cbb_3_*-type terminal oxidase) and NO_3_^–^ respiration. These bacteria are capable of aerobic growth (Kojima and Fukui, 2014; Lin *et al.*, 2021). Their genomic and physiological traits suggest that these bacteria play a role in anammox and PN–anammox reactors as O_2_ scavengers. Notably, these bacteria are able to grow by NO_3_^–^ reduction, and *S. sediminicola* and *Z. arvi* cells reduced NO_3_^–^ to dinitrogen (N_2_) gas (Kojima and Fukui, 2014) and NO_2_^–^ (Lin *et al.*, 2021), respectively, with the potential to produce nitric oxide (NO), as suggested from their metabolic potential (Fig. S2). Therefore, *Zeimonas* sp. ASV884 provides additional NO_2_^–^/NO to anammox bacteria through partial denitrification (Sumino *et al.*, 2006; Waki *et al.*, 2013; Du *et al.*, 2015). *S. sediminicola* and *Z. arvi* utilized a number of carbon sources (*e.g.*, acetate, lactate, propionate, and pyruvate for *S. sediminicola* and L-arabinose, citric acid, L-malic acid, and sodium butyrate for *Z. arvi*) and also H_2_ for NO_3_^–^ reduction (Kojima and Fukui, 2014; Lin *et al.*, 2021). Although the anammox and PN–anammox reactors were operated with inorganic media, organic matter and H_2_ may have been available in the biomass, generated through the degradation of EPS and/or cell debris and fermentative reactions mediated by coexisting *Chloroflexota* bacteria (Kindaichi *et al.*, 2012; Bovio-Winkler *et al.*, 2023). However, the metabolic potentials and interactions in the anammox biomass need to be further exami­ned using cultivation-based ana­lyses in future studies due to the current limitations of metabolic profile predictions using 16S rRNA gene amplicon data (Sun *et al.*, 2020; Toole *et al.*, 2021).

The above core genera coexisting with *Jettenia* sp. ASV326 have been detected in other lab-scale anammox bioreactors (*e.g.*, *Zeimonas* bacteria from a semi–continuous stirred tank reactor) (Ude *et al.*, 2023), while the distribution of these bacteria in wastewater treatment plants (WWTPs) has not yet been investigated. In the present study, the distribution of core ASVs was assessed using the MiDAS4 database, which contains full-length 16S rRNA gene sequences obtained from >740 WWTPs worldwide (Dueholm *et al.*, 2022). The distribution and abundance of *Sulfurisoma*-, *Zeimonas*-, *Phycisphaerales-*, and *Anaerolineae*-related ASVs, which exhibit more than 97% sequence similarity with MiDAS V4 ASV (Table S2), were exami­ned from the MiDAS4 database. The *Sulfurisoma*-related ASV was abundant (mean; 0.52%, *n*=651) and widespread in WWTPs (Fig. 2). The relative abundance of *Sulfurisoma*-related ASV was significantly higher in WWTPs operated for denitrification (the C, N, and DN types in Fig. 2). The higher abundance in WWTPs operated for denitrification suggests the involvement of *Sulfurisoma*-related bacteria in nitrogen removal (*i.e.*, denitrification) in WWTPs, which is consistent with the findings of a previous MiDAS4 survey showing that *Sulfuritales* bacteria related to the *Sulfurisoma*-related ASV were identified as a common denitrifier (Dueholm *et al.*, 2022). Additionally, the physiological traits of *S. sediminicola* (*i.e.*, NO_3_^–^ reduction to N_2_) support the role of *Sulfurisoma*-related bacteria in denitrification (Kojima and Fukui, 2014). On the other hand, no distinct correlation was found between the abundance of *Sulfurisoma*-related ASV and temperature (Fig. 2), suggesting that *Sulfurisoma*-related bacteria adapt to a broad range of temperatures.

In summary, the microbial community structures of mesophilic and low-temperature anammox and PN–anammox reactors were exami­ned to elucidate their ecological and functional roles in nitrogen removal. *Jettenia* sp. ASV326 was identified as the dominant anammox bacterium, and other core coexisting bacteria, such as *Sulfurisoma* sp. ASV867 and *Zeimonas* sp. ASV884, were suggested to contribute to oxygen scavenging and NO_3_^–^ reduction. Notably, *Sulfurisoma*-related bacteria are widely distributed in full-scale WWTPs globally, particularly in those operated for denitrification, where they may contribute to nitrogen removal. These results provide insights into microbial consortia that contribute to biological nitrogen removal in PN–anammox processes.

## Data availability

The raw sequence reads of 16S rRNA gene amplicons are available in the DDBJ nucleotide sequence database under the accession number PRJDB18291.

## Citation

Oshiki, M., Takahashi, K., Kawasaki, S., Choi, H., Park, J., Kim, K., et al. (2025) Microbial Community Structure of Mesophilic and Low-temperature Partial Nitrification-anammox Reactors: Distribution and Functional Roles of the Core Microbiome. *Microbes Environ ***40**: ME25001.

https://doi.org/10.1264/jsme2.ME25001

## Supplementary Material

Supplementary Material

## Figures and Tables

**Fig. 1.**
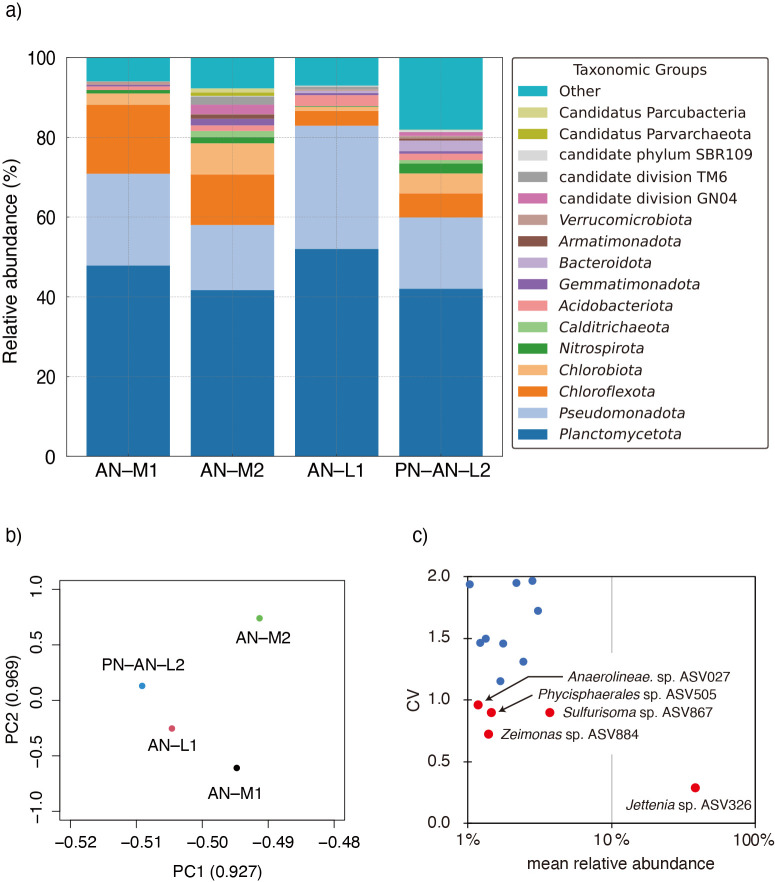
Microbial community structure and core microbiome in partial nitrification (PN)-anammox bioreactors. AN–M1 and AN–M2: mesophilic anammox bioreactors; AN–L1: anammox bioreactor operated at 10°C; PN–AN–L2: PN-anammox bioreactor operated at 7°C (Table 1). **(a)** Relative abundance (%) of 16S rRNA gene reads at the phylum level in each bioreactor. **(b)** Similarity of microbial community structures based on a principal component ana­lysis (PCA) performed using R software (version 4.2.0). The cumulative contributions of the PC1 and PC2 axes were 92.7 and 96.9%, respectively. **(c)** The core microbiome identified based on the mean relative abundance and coefficient of variation (CV) values. Phylogenetic affiliations of ASVs are shown in Fig. S1.

**Fig. 2.**
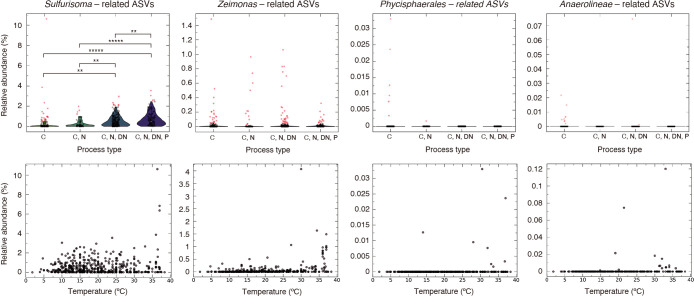
Global distribution of core bacteria associated with anammox bacteria in wastewater treatment plants (WWTPs). The MiDAS4 database, based on a worldwide survey of more than 740 WWTPs using full-length 16S rRNA gene sequences (Dueholm *et al.*, 2022), was analyzed to examine the distribution of core bacteria. The distribution of *Sulfurisoma*-, *Zeimonas*-, *Phycisphaerales*-, and *Anaerolineae*-related ASVs (refer to Table S2 for a list of ASVs) grouped by process types (**upper panel**) and as a function of temperature (**bottom panel**). Process types in the MiDAS4 database are classified as C (WWTPs for carbon removal, *e.g.*, biological oxygen demand, BOD), N (WWTPs for nitrification), DN (WWTPs for denitrification), and P (WWTPs for phosphorus removal). Black plots represent the relative abundance of ASVs in the WWTP samples analyzed from the MiDAS4 database, while red plots denote outliers. Mean values were compared using Welch’s *t*-test corrected with the Bonferroni-Holm method. Asterisks indicate significant differences in the means (**: *P*<0.01; *****: *P*<0.00001).

**Table 1.** Operational conditions and nitrogen removal performance of anammox and partial nitrification (PN)–anammox reactors. The compositions of the inorganic synthetic wastewater supplied to the bioreactors are provided in Table S1.

	AN–M1 (anammox) Sequencing batch reactor	AN–M2 (anammox) Upflow granular reactor	AN–L1 (anammox) Upflow granular reactor	PN–AN–L2 (PN–anammox) Baffled reactor
Temperature	33°C	37°C	10°C	7°C
Volume	10 L	22 L	1.1 L	5 L
Influent pH	7.7	7.4	7.7	7.5
Dissolved oxygen (mg O_2_ L^–1^)	n.a.	n.a.	n.a.	<0.5
Hydraulic retention time (h)	24	24	2	4
Nitrogen loading rates (kg N m^–3^ d^–1^)	0.23	0.38	0.84	0.26
Nitrogen removal rates (kg N m^–3^ d^–1^)	0.20	0.32	0.67	0.16
Nitrogen removal efficiency	87%	84%	79%	60%
Influent (mg N L^–1^)				
NH_4_^+^	100	175	30	40
NO_2_^–^	130	231	40	—
Effluent (mg N L^–1^)				
NH_4_^+^	n.d.	9.3	n.d.	10
NO_2_^–^	n.d.	6.6	4.9	n.d.
NO_3_^–^	28.9	44.2	7.7	6.99

n.a.; not applicable, n.d.; not detected
