# Supplementary material for: Microbial Community Structure of Mesophilic and Low-temperature Partial Nitrification-anammox Reactors: Distribution and Functional Roles of the Core Microbiome
Source: Microbes Environ. 2025 Apr 26;40(2):ME25001. doi: 10.1264/jsme2.ME25001 (PMC12213063; doi:10.1264/jsme2.ME25001)
Supplement: Supplementary file 1 — Supplementary Material [file 40_25001_s1.pdf]

Supplementary information (ME25001)

Microbial Community Structure of Mesophilic and Low-temperature Partial Nitrification-anammox Reactors: Distribution and Functional Roles of the Core Microbiome

Mamoru Oshiki[1*], Kohei Takahashi[1,2], Seiya Kawasaki[1], Hyungmin Choi[3], Jihye Park[4], Kwiyong Kim[3,5], Hyokwan Bae[3,5], Satoshi Okabe[1], and Changsoo Lee[3,5]

[1]*Division of Environmental Engineering, Faculty of Engineering, Hokkaido University, Sapporo, Japan.*

[2]*JSPS Postdoctoral Fellow, Hokkaido University, Japan.*

[3]*Department of Civil Urban Earth and Environmental Engineering, Ulsan National Institute of Science and Technology (UNIST), Republic of Korea.*

[4]*Department of Civil and Environmental Engineering, Pusan National University, Republic of Korea.*

[5]*Graduate School of Carbon Neutrality, Ulsan National Institute of Science and Technology (UNIST), Republic of Korea.*

Table S1. The compositions of the inorganic synthetic wastewater supplied to the bioreactors.
PN; partial nitrification, SBR; sequencing batch reactor, TES; trace element solution.

AN–M1 (anammox) SBR	AN–M2 (anammox) Upflow granular reactor	AN–L1 (anammox) Upflow granular reactor	PN–AN–L2 (PN-anammox) Baffled reactor
100 mg–N/L as $(NH_4)_2SO_4$	175 mg–N/L as NH_4Cl	30 mg–N/L as $(NH_4)_2SO_4$	40 mg–N/L as NH_4Cl
130 mg–N/L as $NaNO_2$	231 mg–N/L as $NaNO_2$	39.6 mg–N/L as $NaNO_2$	69 mg C/L as $NaHCO_3$
80 mg C/L as $NaHCO_3$	95 mg C/L as $NaHCO_3$	80 mg C/L as $NaHCO_3$	6 mg P/L as KH_2PO_4
6 mg P/L as KH_2PO_4	6 mg P/L as KH_2PO_4	6 mg P/L as KH_2PO_4	12 mg Mg/L as $MgSO_4\cdot 7H_2O$
12 mg Mg/L as $MgSO_4\cdot 7H_2O$	12 mg Mg/L as $MgSO_4\cdot 7H_2O$	12 mg Mg/L as $MgSO_4\cdot 7H_2O$	48 mg Ca/L as $CaCl_2\cdot 2H_2O$
48 mg Ca/L as $CaCl_2\cdot 2H_2O$	48 mg Ca/L as $CaCl_2\cdot 2H_2O$	48 mg Ca/L as $CaCl_2\cdot 2H_2O$	1 mL/L TES III[c]
1 mL/L TES I	1 mL/L TES III	1 mL/L TES I	1 mL/L TES IV
1 mL/L TES II	1 mL/L TES IV	1 mL/L TES II	

TES I (per liter): 5 g $EDTA\cdot 2H_2O$ and 5 g $FeSO_4\cdot 7H_2O$

TES II (per liter): 0.22 g $Na_2MoO_4\cdot 2H_2O$, 0.014 g H_3BO_3, 0.19 g $NiCl_2\cdot 6H_2O$, 0.108 g Na_2SeO_4, 0.43 g $ZnSO_4\cdot 7H_2O$, 0.24 g $CoCl_2\cdot 6H_2O$, 0.99 g $MnCl_2\cdot 4H_2O$, and 0.25 g $CuSO_4\cdot 5H_2O$

TES III (per liter): 5 g EDTA and 22 g $FeCl_2$

TES IV (per liter): 10 g EDTA, 0.22 g $Na_2MoO_4\cdot 2H_2O$, 0.014 g H_3BO_3, 0.19 g $NiCl_2\cdot 6H_2O$, 0.21 g $Na_2SeO_4\cdot 10H_2O$, 0.43 g $ZnSO_4\cdot 7H_2O$, 0.24 g $CoCl_2\cdot 6H_2O$, 0.99 g $MnCl_2\cdot 4H_2O$, and 0.25 g $CuSO_4\cdot 5H_2O$

Table S2. A list of MiDAS ASV IDs with >97% sequence identity to the ASVs detected from partial nitrification–anammox bioreactors. The 16S rRNA gene sequences of *Sulfurisoma* sp. ASV867, *Zeimonas* sp. ASV884, *Phycisphaerales* sp. ASV505, and *Anaerolineae* sp. ASV027 were subjected to a blastn search against the MiDAS 4 database.

Sulfurisoma-related ASV	*Zeimonas*-related ASV	*Phycisphaerales*-related ASV	*Anaerolineae*-related ASV
ASV135	ASV1029	ASV18963	ASV20019
ASV212	ASV1299		ASV24954
ASV274	ASV1750		ASV29147
ASV309	ASV3133		
ASV378	ASV4097		
ASV532	ASV5436		
ASV621	ASV7804		
ASV649	ASV15110		
ASV695	ASV16691		
ASV728	ASV22765		
ASV876	ASV23850		
ASV1492	ASV28022		
ASV1557	ASV37920		
ASV1825			
ASV1987			
ASV2127			
ASV3234			
ASV4056			
ASV11716			
ASV12725			
ASV16928			
ASV20187			
ASV21427			
ASV29379			
ASV36287			

Figure legends for supplementary figures.

Figure S1. Phylogeny and abundance of 16S rRNA gene amplicon sequence variants (ASVs) in anammox and partial nitrification (PN)-anammox bioreactors. The 16S rRNA gene reads obtained from amplicon sequencing were clustered into amplicon sequence variants (ASVs), and a phylogenetic tree was constructed using MEGA11 software. The tree was calculated using the neighbor-joining (Maximum composite likelihood model, 1,000 iterations), maximum likelihood (Tamura-Nei model, 200 iterations), and maximum parsimony (Subtree-pruning-regrafting model, 500 iterations) methods. *Sulfuritalea hydrogenivorans* (accession number AP012547.1) was used as the outgroup. Branching points with bootstrap support probabilities >80% are indicated by filled circles. The scale bar represents 8% sequence divergence. The heatmap illustrates the relative abundance of each ASV in the total biomass. AN–M1 and AN–M2: mesophilic anammox bioreactors; AN–L1: anammox bioreactor operated at 10°C; PN–AN–L2: PN-anammox bioreactor operated at 7°C (See **Table 1** for detail).

Figure S2. Metabolic potentials of the core microbiome and aerobic ammonia- and nitrite-oxidizing bacterial amplicon sequence variants (ASVs). Metabolic potentials were analyzed using PICRUSt2 software and manually annotated with closely related species genomes using KAAS and DRAM annotation tools. The heatmap represents the completeness of metabolic pathways, calculated using the KEGG decoder.

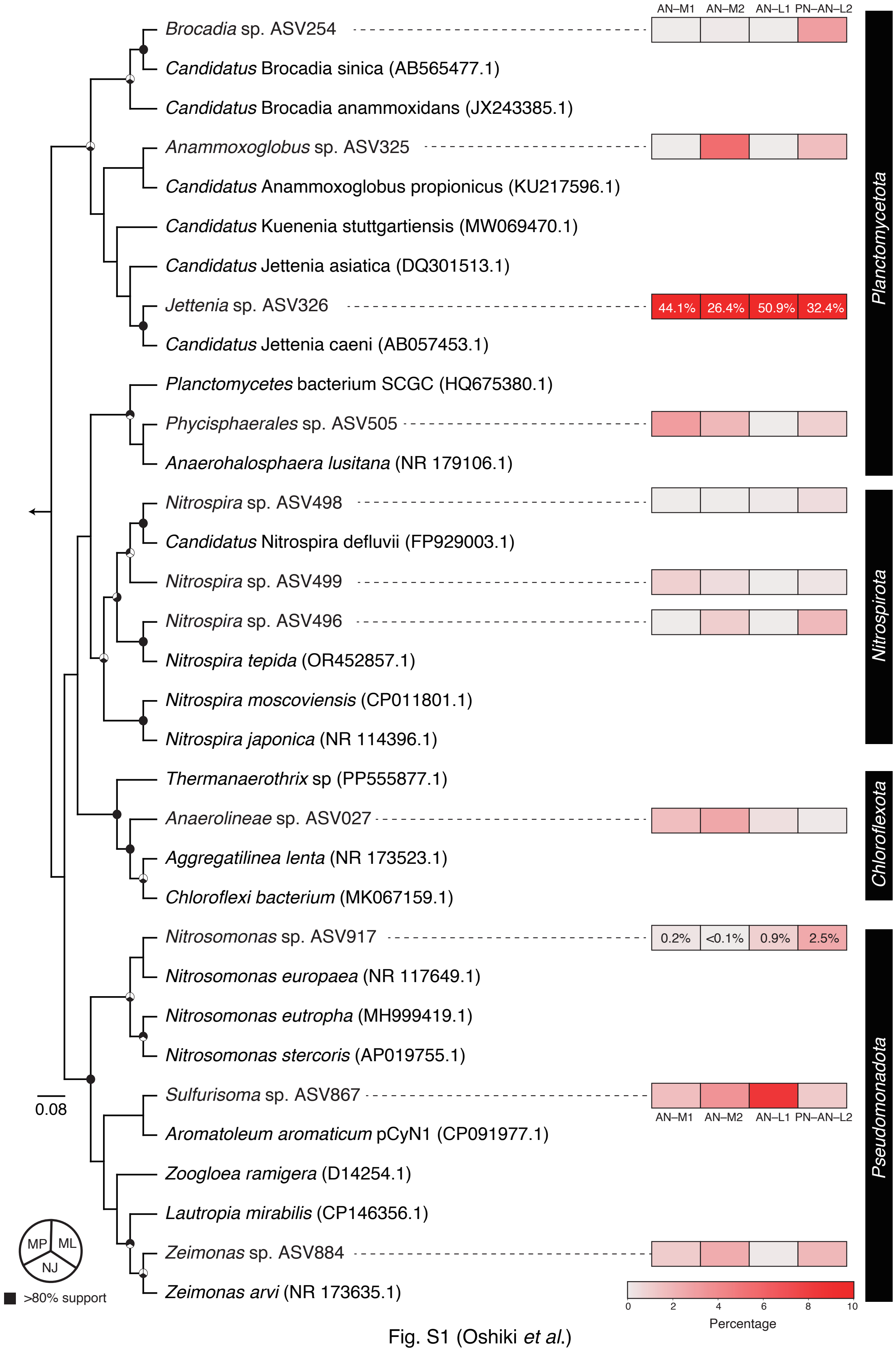

Fig. S1 (Oshiki *et al.*)

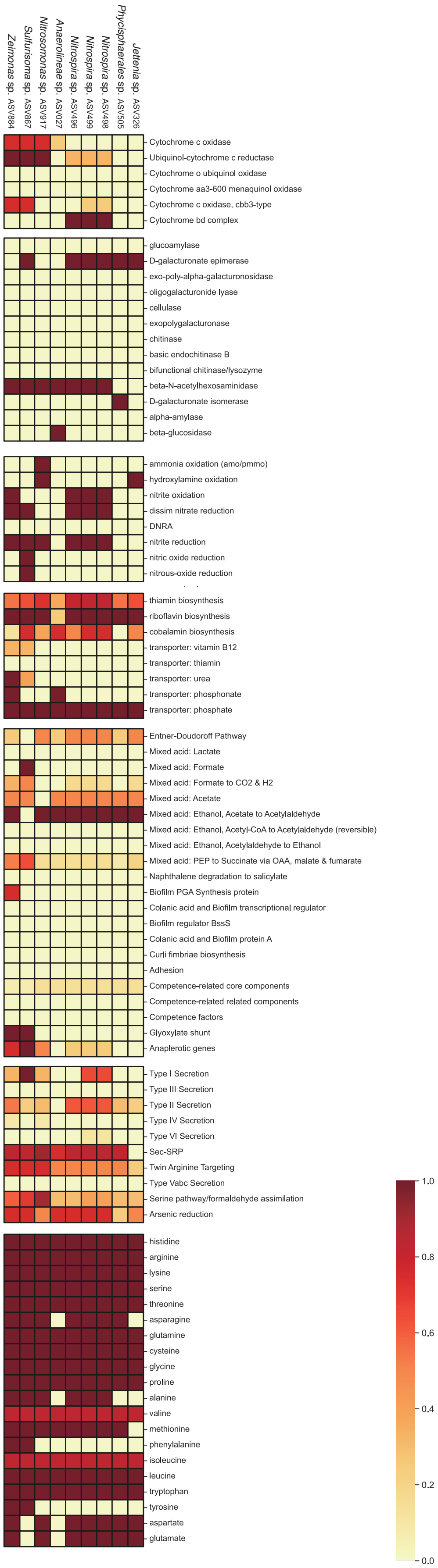

Fig. S2 (Oshiki *et al.*)
